# Cardiovascular Phenotypes Profiling for L-Transposition of the Great Arteries and Prognosis Analysis

**DOI:** 10.3389/fcvm.2021.781041

**Published:** 2022-01-21

**Authors:** Qiyu He, Huayan Shen, Xinyang Shao, Wen Chen, Yafeng Wu, Rui Liu, Shoujun Li, Zhou Zhou

**Affiliations:** ^1^Pediatric Cardiac Center, Fuwai Hospital, Chinese Academy of Medical Sciences and Peking Union Medical College, Beijing, China; ^2^Department of Laboratory Medicine, National Center for Cardiovascular Diseases, Fuwai Hospital, Chinese Academy of Medical Sciences and Peking Union Medical College, Beijing, China; ^3^Center for Applied Statistics, School of Statistics, Renmin University of China, Beijing, China

**Keywords:** congenitally corrected transposition of the great arteries, human phenotype ontology, surgery, risk stratification, prognosis

## Abstract

**Objectives:**

Congenitally corrected transposition of the great arteries (ccTGA) is a rare and complex congenital heart disease with the characteristics of double discordance. Enormous co-existed anomalies are the culprit of prognosis evaluation and clinical decision. We aim at delineating a novel ccTGA clustering modality under human phenotype ontology (HPO) instruction and elucidating the relationship between phenotypes and prognosis in patients with ccTGA.

**Methods:**

A retrospective review of 270 patients diagnosed with ccTGA in Fuwai hospital from 2009 to 2020 and cross-sectional follow-up were performed. HPO-instructed clustering method was administered in ccTGA risk stratification. Kaplan-Meier survival, Landmark analysis, and cox regression analysis were used to investigate the difference of outcomes among clusters.

**Results:**

The median follow-up time was 4.29 (2.07–7.37) years. A total of three distinct phenotypic clusters were obtained after HPO-instructed clustering with 21 in cluster 1, 136 in cluster 2, and 113 in cluster 3. Landmark analysis revealed significantly worse mid-term outcomes in all-cause mortality (*p* = 0.021) and composite endpoints (*p* = 0.004) of cluster 3 in comparison with cluster 1 and cluster 2. Multivariate analysis indicated that pulmonary arterial hypertension (PAH), atrioventricular septal defect (AVSD), and arrhythmia were risk factors for composite endpoints. Moreover, the surgical treatment was significantly different among the three groups (*p* < 0.001) and surgical strategies had different effects on the prognosis of the different phenotypic clusters.

**Conclusions:**

Human phenotype ontology-instructed clustering can be a potentially powerful tool for phenotypic risk stratification in patients with complex congenital heart diseases, which may improve prognosis prediction and clinical decision.

## Introduction

Congenitally corrected transposition of the great arteries (ccTGA), a rare and anatomically complex congenital heart disease with an incidence of 1 in 33,000 live births approximately, is characterized by atrioventricular and ventricular-arterial discordance (1). Diverse accompanied anomalies are ubiquitous that the most common co-deformities are ventricular septal defect (VSD, 70%), pulmonary stenosis (40%), and systemic atrioventricular valve abnormality (2). Heterogeneous physiological conditions and hemodynamic issues accrue, thus, impede clinical decisions and prognostic evaluation.

The human phenotype ontology (HPO), a comprehensive resource for systematically defining and logically organizing human phenotypes, enables computational inference and complex algorithms that support combinatorial genomic and phenotypic analysis (3). It has been used in multiple fields, particularly, genotype-phenotype analysis for genetic syndromes (4), neurodevelopmental diseases (5–7), hereditary hemorrhagic telangiectasia (8), and myofibrillar myopathies (9). Moreover, it is appealing that HPO is adopted as a powerful tool for personalized medicine and precision medicine (3, 10). The scope of HPO application has gradually broadened as HPO-related procedures were evolved, such as Doc2Hpo (11) for HPO concept curation and HPOLabeler (12) for human protein–phenotype studies. Combined with electronic medical records (EMRs), HPO can be administered in constructing longitudinal footprints of genetic disorders (13) and expediting genetic diagnoses (14). Previously, we have succeeded in grouping patients with Ebstein's anomaly by employing HPO and EMR (15).

There had been several reports investigating the postoperative outcomes of patients with ccTGA that most of them were complicated with VSD and pulmonary stenosis (or left ventricular outflow tract obstruction) (16, 17). However, it seemed to be a common problem that most studies did not take all of the cardiovascular phenotypes into consideration for prognostic analysis, thus bias might exist. Here, we delineated a big cardiovascular phenotypes picture of 270 patients with ccTGA, clustered them according to phenotypic similarity by HPO and EMR, and analyzed the outcomes in combination with three types of surgical strategies. We aimed at elucidating the relationship between phenotype and prognosis and providing a novel phenotypic stratification strategy that might improve prognosis prediction and clinical decision of ccTGA.

## Patients and Methods

### Study Population

By retrospectively reviewing records from 2009 to 2020, we identified 380 patients who were admitted to Fuwai hospital and underwent surgeries with the diagnosis of ccTGA by Chinese EMR. The international classification of diseases 10th revision (ICD-10) has been adopted in our center and all diagnoses in Chinese can be referred to this system. Standardized diagnosis in ICD-10 was further annotated based on HPO. The diagnoses of all enrolled patients were confirmed by echocardiography and surgery. Baseline demographics, echocardiographic information, electrocardiographs, cardiac CT, catheter data, operation and progress notes, and status at discharge were manually reviewed by two authors and a specialized cardiologist. There was no disagreement in the EMR reviewing process. Then we excluded complex congenital heart disease (pulmonary atresia, single ventricle, and double outlet right ventricle) which might skew our clustering results, thus making ccTGA the main diagnosis. A total of 300 patients were included in further analysis. This study was approved by the Ethics committee of Fuwai Hospital, Chinese Academy of Medical Science, and Peking Union Medical College (approval no. 2020-1402). Owing to the nature of the retrospective study, informed consent was waived. The whole process of this study is displayed in Supplementary Figure 1.

### Phenotype Annotation Based on HPO

We collated cardiovascular phenotypes based on surgical and echocardiographic records (before definitive surgery) and standardized them in concordance with the HPO database (https://hpo.jax.org/). All included terms were the subitems of “HP:0001626, abnormality of the cardiovascular system” except for “HP:0002092, pulmonary arterial hypertension” (PAH). Phenotypes that did not appear in the HPO database would be traced to superior terms, for example, “junctional escape rhythm” was taken as “arrhythmia.” Collectively, a total of 58 terms were annotated.

### Patients Clustering by HPO

The principles of phenotypes clustering based on HPO were as previously described (15, 18–20). Our subsequent clustering was initially based on calculating the similarity between any two annotated phenotypes. According to the frequency information of each phenotype in the HPO dataset [p(p)], we defined the similarity of a pair of phenotypes (e.g., p1 and p2) as follows:


$$\begin{array}{l} Sim\;\left(p1,\;p2\right)\;=\underset{v\in anc\left(p1\right)\cap anc\left(p2\right)}{\max}\;-logp\left(v\right)\; \end{array}$$

$$\begin{array}{l} v\in anc\left(p1\right)\cap anc\left(p2\right):\text{the set of common ancestors of p}1\text{ and p}2. \end{array}$$

Patients are defined by a set of phenotypes, we calculated the similarity matrix in pairwise patients (e.g., c1 and c2) based on “between term set” similarities by the following equation:


$$\begin{array}{l} sim\left(c1,\;c2\right)=\frac{1}{2|c_{1}|}\underset{p1\in c1}{\sum }\underset{p2\in c2}{\max}sim\left(p1,p2\right)+\; \end{array}$$

$$\begin{array}{l} \frac{1}{2|c_{2}|}\underset{p1\in c2}{\sum }\underset{p2\in c1}{\max}sim\left(p1,p2\right)\; \end{array}$$

Guided by R package “ontologySimilarity” and based on similarity matrix (sim_mat), we calculated a distance matrix [max(sim_mat) – sim_mat]. Using the distance matrix, we performed an unsupervised hierarchical clustering by R package “pheatmap.” For the selection of the parameter in the function “pheatmap,” the complete linkage method was employed by default, and “cutree_col” is set to be three to obtain three phenotypically heterogeneous clusters (https://cran.r-project.org/web/packages/ontologyIndex/vignettes/intro-to-ontologyX.html, https://rdrr.io/cran/ontologySimilarity/f/vignettes/ontologySimilarity-examples.Rmd).

### Follow-Up and Clinical Adverse Outcomes

The records of patients who received re-examinations in our center were retrieved (such as ECG, echocardiography, any records of readmission, or reported adverse events). A follow-up telephone interview for all enrolled patients was also conducted on March 2021 (inquiring about their survival status, morbidity, any adverse events, or reintervention), and revisiting records from other hospitals were obtained if available. Total clinical adverse events included all-cause mortality, heart failure, and reinterventions. Primary surgery was defined as definitely corrective surgery and reintervention was any heart surgery performed after the primary surgery. We treated all-cause mortality as the primary endpoint, and all-cause mortality plus heart failure as the composite endpoint events. For the patients who could not be reached, the last medical visit records in our hospital were retrieved as the basis for outcome judgment.

### Surgical Classification

Due to diverse surgical procedures among the patients, we classified them into 3 types and were approximately consistent with a recent study (21).

- Anatomic repair (arterial switch/double arterial root switch/Rastelli with Senning or Mustard, arterial switch/Rastelli with Hemi-Mustard, and bidirectional Glenn).
- Physiologic repair (any cardiac surgery other than anatomical repair and permanent pacemaker placement, thus morphological right ventricle remains as the systemic ventricle).
- Fontan palliation.

### Statistical Analysis

All statistical analyses involved in this study were performed with SPSS Statistics Version 23.0 (IBM 16 Corporation, Armonk, NY, USA) and R software version 3.6.2 (R Foundation for Statistical Computing, Vienna, Austria). Categorical variables were summarized as frequencies (percentage) and continuous variables were summarized as mean ± SD or median (25th to 75th percentiles), with the comparison methods of χ^2^ test unless group size was lesser than 10, in which the Fisher exact test and Kruskal-Wallis test would be adopted.

Kaplan-Meier method was adopted to estimate the freedom from adverse events morbidity, and overall survivals in which log-rank test was administered in the comparison between different groups. The survival time of enrolled patients began at the definitive surgery and ended at death, event, or the last follow-up. Stratification in Kaplan-Meier analysis was based on different clusters to detect whether significance existed or not. Stratification based on different surgical strategies was performed to explore the influence of different surgical types on the novel clustering modality. Moreover, the mid- to long-term outcome is the main issue in patients with ccTGA. In this regard, landmark analysis was administered for piecewise analysis of patient outcomes. To identify underlying associated factors' correlation or contribution to adverse events and overall survival rate, the univariable Cox proportional hazard regression method was utilized. After stepwise selection of potential variables, we adopted the multivariable Cox proportional hazard regression method to further validate the significant univariate factors (*p* < 0.05). We used the Benjamini-Hochberg method to adjust the *p*.

## Results

### Patient Demographics and Characteristics

Baseline demographic characteristics of this cohort are illustrated in Table 1. The median age at definitive surgery was 5.4 years (2.1–23.25 years), with 107 female patients (39.6%). The mean follow-up time was 4.88 ± 3.47 years, the median follow-up time was 4.29 (2.07–7.37) years. Thirty patients were lost to follow-up and the follow-up rate was reached 90% (270/300). Clinical adverse events occurred in a total of 48 patients of which 19 patients died of various causes, 7 patients had heart failure, and 28 patients received reinterventions. Table 1 shows the co-existed anomalies presented in more than 10 patients. VSD, tricuspid regurgitation (TR), and pulmonic stenosis (PVS) were the top three concomitant defects with the frequency of 80.8, 47.4, and 42.6%, respectively. Preoperative systemic ventricular ejection fraction (SVEF) and systemic ventricular end diastolic diameter (SVEDD) were significantly different among three clusters with a *p* of 0.002 and 0.001, respectively. However, patients with SVEF <40% among the three clusters were comparable (*p* = 0.082). Notably, the occurrence of TR, the most concerning issue of ccTGA, was significantly diverse among three clusters (*p* < 0.001) with the severity demonstrated in Table 1.

**Table 1:** Baseline demographics of patients at definitive surgery and follow-up.

**Variables**	**All cohort (*n* = 270)**	**Cluster 1 (*n* = 21)**	**Cluster 2 (*n* = 136)**	**Cluster 3 (*n* = 113)**	***P*-value**
Age at definitive surgery, years	13.73 ± 15.78 5.4 (2.1–23.25)	32.50 ± 18.61 31 (20.00–49.00)	10.36 ± 12.33 5.00 (2.00–14.00)	14.29 ± 16.51 5.30 (2.00–24.00)	<0.001^*^
Age at follow-up, years	20.63 ± 15.85 14.40 (9.20–31.03)	39.21 ± 18.51 42.30 (25.85–55.35)	17.67 ± 12.44 13.70 (9.25–22.48)	20.75 ± 16.74 13.90 (8.70–31.75)	<0.001^*^
Follow-up, years	4.88 ± 3.47 4.29 (2.07–7.37)	5.91 ± 3.98 4.88 (2.87–7.56)	4.88 ± 3.64 4.31 (1.96–7.59)	4.68 ± 3.14 4.10 (1.99–7.32)	0.452
Female (n, %)	107 (39.6%)	9 (42.9%)	52 (38.2%)	46 (40.7%)	0.879
BMI	17.27 ± 3.69 16.30 (14.78–19.14)	21.85 ± 4.72 21.11 (17.95–25.54)	16.89 ± 3.54 15.74 (14.71–18.60)	16.90 ± 3.10 16.39 (14.61–18.78)	<0.001^*^
Surgical strategy					<0.001^*^
Anatomical repair	68 (25.2%)	1 (4.8%)	39 (28.7%)	28 (24.8%)	
Physiological repair	153 (56.7%)	19 (90.5%)	63 (46.3%)	71 (62.8%)	
Fontan	47 (17.4%)	0	34 (25.0%)	13 (11.5%)	
Associated anomalies					
ASD	82 (30.31%)	1 (4.8%)	54 (39.7%)	27 (23.9%)	0.001^*^
VSD	194 (80.8%)	0	127 (93.4%)	67 (59.3%)	<0.001^*^
POF	48 (17.8%)	0	31 (22.8%)	17 (15.0%)	0.024^*^
PDA	18 (6.7%)	1 (4.8%)	8 (5.9%)	9 (8.0%)	0.755
PVS	115 (42.6%)	1 (4.8%)	92 (67.6%)	22 (19.5%)	<0.001^*^
TR	128 (47.4%)	16 (76.2%)	45 (33.1%)	67 (59.3%)	<0.001^*^
MR	40 (14.8%)	6 (28.6%)	16 (11.8%)	18 (15.9%)	0.119
PS	17 (6.3%)	1 (4.8%)	1 (0.7%)	15 (13.3%)	<0.001^*^
LSVC	12 (4.4%)	1 (4.8%)	3 (2.2%)	8 (7.1%)	0.178
RAA	11 (4.1%)	0	5 (3.7%)	6 (5.3%)	0.499
ALVOT	10 (3.7%)	0	7 (5.1%)	3 (2.7%)	0.377
Cardiomegaly	41 (15.2%)	10 (47.6%)	9 (6.6%)	22 (19.5%)	<0.001^*^
PAH	40 (14.8%)	8 (38.1%)	0	32 (28.3%)	<0.001^*^
Mild	10 (3.7%)	4 (19.0%)		6 (5.3%)	
Intermediate	16 (5.9%)	3 (14.3%)		13 (14.2%)	
Severe	14 (5.2%)	1 (4.8%)		13 (14.2%)	
Cardiac malposition	60 (22.2%)	4 (19.0%)	35 (25.7%)	21 (18.6%)	<0.001^*^
Dextrocardia	37 (13.7%)	4 (19.0%)	18 (13.2%)	15 (13.3%)	
Mesocardia	11 (4.1%)	0	8 (5.9%)	3 (2.7%)	
Levocardia	12 (4.4%)	0	9 (6.6%)	3 (2.7%)	
Arrhythmia	35 (12.9%)	7 (33.3%)	11 (8.1%)	17 (15.0%)	0.018^*^
AVB	21 (7.8%)	2 (9.5%)	10 (7.4%)	9 (8.0%)	
AF	8 (3.0%)	5 (23.8%)	1 (0.7%)	2 (1.8%)	
VE	5 (1.9%)	3 (14.3%)	0	2 (1.8%)	
Others	17 (6.3%)	8 (38.1%)	1 (0.7%)	8 (7.1%)	
SAVV regurgitation	128 (47.4%)	16 (76.2%)	45 (33.1%)	67 (59.3%)	<0.001^*^
Mild	15 (5.6%)	0	7 (5.1%)	8 (7.1%)	
Moderate	5 (1.9%)	1 (4.8%)	0	4 (3.5%)	
Severe	108 (40.0%)	15 (71.4%)	38 (27.9%)	55 (48.7%)	
SVEF (%) at definitive surgery	61.21 ± 7.51 60.00 (58.00–65.00)	54.64 ± 10.77 58.00 (51.00–60.00)	62.05 ± 6.34 61.80 (60.00–65.00)	61.34 ± 7.67 60.00 (58.00–65.00)	0.002^*^
SVEF <40% at definitive surgery	5 (1.9%)	2 (9.5%)	1 (0.7%)	2 (1.8%)	0.082
SVEF (%) at follow-up	58.09 ± 9.86 60.00 (55.00–64.00)	51.67 ± 11.80 58.00 (45.00–60.00)	58.93 ± 9.47 60.00 (58.50–65.00)	58.06 ± 9.76 60.00 (55.00–64.25)	0.033^*^
SVEF < 40% at follow-up	20 (7.4%)	3 (14.2%)	9 (6.6%)	8 (7.1%)	<0.001^*^
SVEDD (mm) at definitive surgery	34.72 ± 13.96 32.00 (25.00–41.50)	50.17 ± 19.82 59.50 (29.25–65.25)	31.95 ± 12.41 29.50 (24.00–38.00)	35.31 ± 12.73 32.00 (25.50–44.50)	0.001^*^
SVEDD (mm) at follow-up	38.18 ± 11.63 37.00 (29.00–46.50)	44.71 ± 10.77 40.50 (37.75–54.00)	36.73 ± 11.68 36.00 (28.00–44.00)	38.78 ± 11.42 38.00 (29.00–49.00)	0.044^*^

*All values presented as mean ± SD, median (25–75th percentiles), or n (%)*.

* *Indicated statistical significance*.

*ALVOT, abnormal left ventricular outflow tract morphology; ARV, abnormal right ventricle morphology; AR, aortic regurgitation; ASD, atrial septal defect; AVC, abnormal vena cava morphology; AVSD, atrioventricular septal defect; BMI, body mass index; BPV, bicuspid pulmonary valve; LSVC, persistent left superior vena cava; MR, mitral regurgitation; PAH, pulmonary arterial hypertension; PDA, patent ductus arteriosus; POF, patent foramen ovale; PR, pulmonary regurgitation; PS, pulmonary artery stenosis; PVS, pulmonary valve stenosis; RAA, right aortic arch; SV, systemic ventricle; SVEDD, systemic ventricular end diastolic diameter; SVEF, systemic ventricular ejection fraction; TR, tricuspid regurgitation; VSD, ventricular septal defect*.

### HPO-Based Clustering for Patients With CcTGA

**Figure 1:**
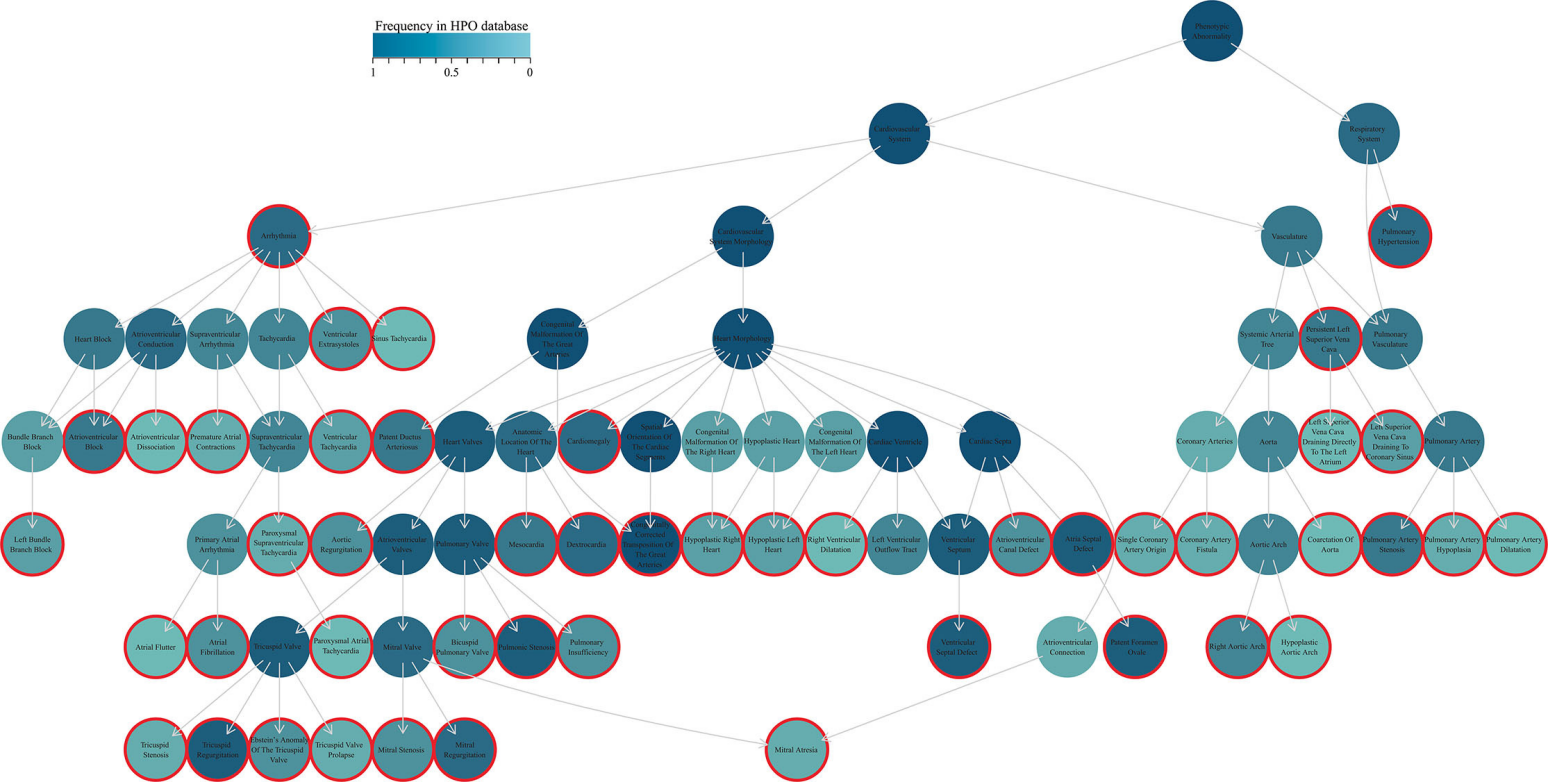
HPO terms encoded for the ccTGA-associated cardiovascular anomalies. The tree plot shows the relationship of all the annotated phenotypes. Circles with borders are the phenotypes presented in our cohort (the phenotypes absent in the HPO database are not shown). The shade of the color represents the frequency of terms in the HPO database (the darker the color, the higher the frequency, with the color key on the top). Arrows indicate the relationship of affiliation between phenotypes. HPO, the human phenotype ontology; ccTGA, congenitally corrected transposition of the great arteries.

**Figure 2:**
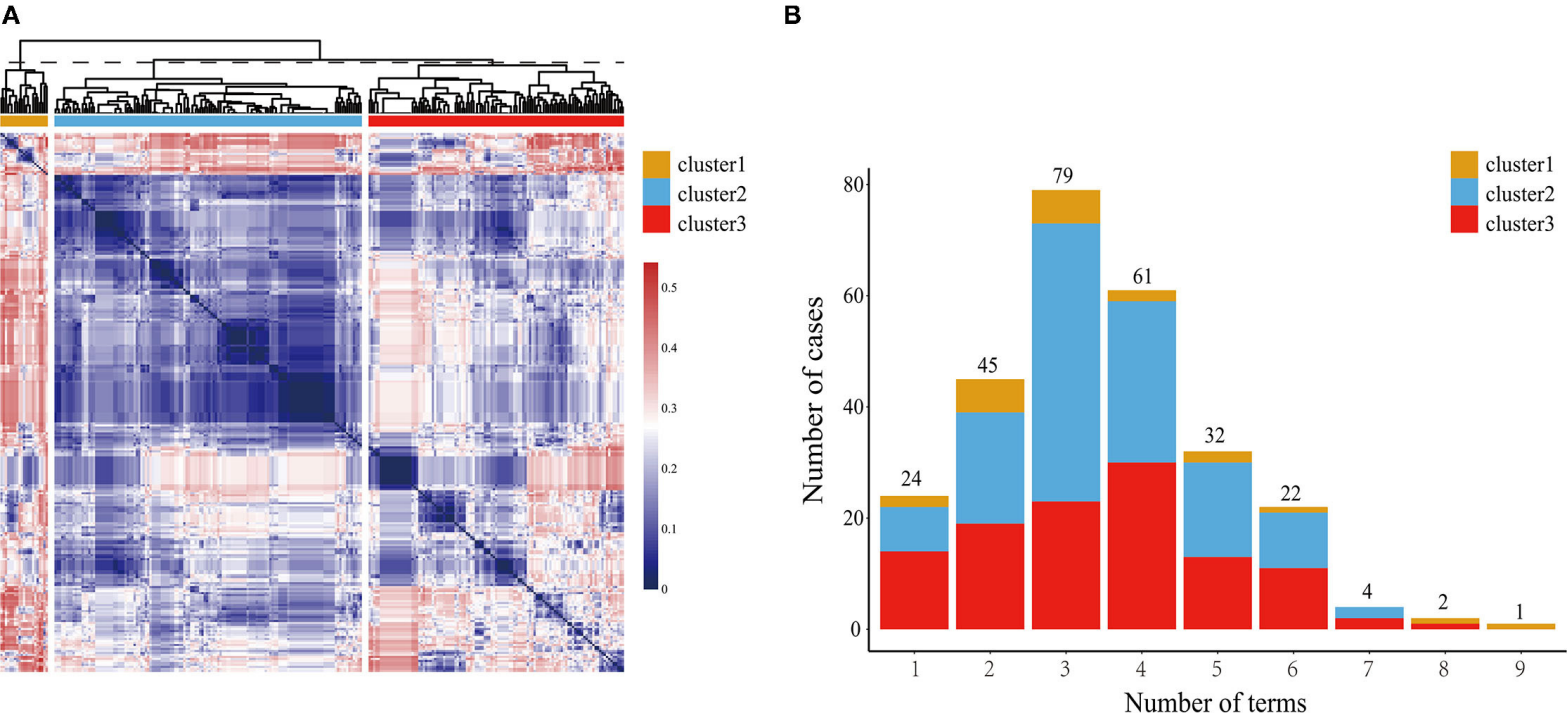
Characteristics of three phenotypic clusters. **(A)** Heatmap of clustering for patients with ccTGA. The phenotypic similarity was calculated to generate the distance matrix, which was further used to produce the heatmap. Both horizontal and vertical axis indicated patients with ccTGA. The dashed line showed the height to cut the tree into three groups. The color represents the degree of similarity between patients (lighter color indicates higher similarity, with the color key on the right) yellow, blue, and red were adopted to distinguish three different clusters (yellow for cluster 1, blue for cluster 2, and red for cluster 3); **(B)** Number of phenotypes of patients in each cluster. ccTGA, congenitally corrected transposition of the great arteries.

We sorted out the cardiovascular phenotypes of all the patients and annotated them to 58 terms. Detailed information of the 58 terms in HPO and the abbreviations assigned in the study are presented in Supplementary Table 1. A tree plot was generated to show the distribution and affiliation of each term, and the shade of the color represented the frequency of each item in the HPO database (Figure 1). Most patients presented three or four additional terms, with the median number of additional terms carried by each patient was three (ranging from one to nine) (Figure 2B).

We then performed an unsupervised hierarchical clustering to classify 270 patients into three clusters based on their phenotypic similarity, with a size of 21, 136, and 113, respectively (Figure 2A). We compared the distribution of 58 terms in three clusters and found that 15 of 58 terms had significant differences in the distribution among the three clusters (Table 1, Supplementary Table 2, Supplementary Figure 2).

Three clusters had their characteristics in phenotypic distribution. Patients in cluster 1 had higher frequencies of TR, PAH, cardiomegaly, and arrhythmia. Septal defects and PVS more frequently occurred in cluster 2. Cluster 3 had no distinct but wide-ranging phenotypic characteristics. Next, we analyzed the phenotypic combination distribution of patients in the three clusters and found that cluster 2 had more homogeneous phenotypes while cluster 1 and cluster 3 were more heterogeneous (Supplementary Figure 3). For cluster 3, more complex phenotypic combinations were observed and a total of 46 isolated terms with 87 different combinations occurred. Seventy-eight patients had a unique phenotypic combination, and the median number of terms carried by each patient in cluster 3 was four compared with cluster 1 and cluster 2 with the median number of three (Figure 2B).

### Clinical Outcomes

First, we summarized the overall prognosis of the whole cohort. The overall 30-day survival rate is 99.26% (95% CI: 98.24–100%), the 5-year survival rate is 94.29% (95% CI: 91.11–97.57%), and the 10-year survival rate is 85.4% (95% CI: 78.59–92.79%), with detail information documented in Supplementary Table 3. We performed a univariable Cox proportional regression analysis for all phenotypes revealing that, PAH, atrioventricular septal defect (AVSD), mitral regurgitation (MR), pulmonary insufficiency (PI), cardiomegaly, levocardia (annotated as ‘HP:0004307, abnormal anatomic location of the heart'), and arrhythmia were risk factors of death and heart failure (Table 2). Among them, PAH, AVSD, and arrhythmia were significantly associated with the occurrence of composite endpoint events in multivariable analysis (Table 2).

**Table 2:** Univariable and multivariable analysis for outcomes.

**Variables**	**Death**			**Composite endpoints**		
	**Hazard ratio (95%CI)**	***P*** **value**	**P-adjust value**	**Hazard ratio (95%CI)**	***P*** **value**	**P-adjust value**
**Univariate**						
**Baseline characteristics**						
Age	0.985 (0.952–1.019)	0.389	0.748	1.003 (0.980–1.027)	0.792	0.997
BMI	0.946 (0.824–1.086)	0.431	0.754	0.916 (0.801–1.047)	0.199	0.445
SVEF	0.989 (0.966–1.014)	0.392	0.748	0.982 (0.964–1.000)	0.053	0.181
SV dysfunction	6.470 (1.478–28.330)	0.013^*^	0.147	5.398 (1.386–21.020)	0.015^*^	0.161
SVEDD	1.008 (0.981–1.035)	0.581	0.849	0.995 (0.970–1.030)	0.972	0.998
PAH	3.649 (1.432–9.301)	0.007^*^	0.123	3.640 (1.603–8.266)	0.002^*^	0.035^*^
**Associated anomalies**						
ASD	0.799 (0.288–2.220)	0.667	0.849	0.958 (0.416–2.210)	0.920	0.998
VSD	1.026 (0.369–2.857)	0.960	0.998	1.282 (0.511–3.213)	0.596	0.869
AVSD	18.920 (5.255–68.110)	<0.001^*^	0.035^*^	12.980 (3.767–44.760)	<0.001^*^	0.035^*^
POF	2.223 (0.844–5.855)	0.106	0.464	1.856 (0.775–4.447)	0.165	0.413
PDA	1.045 (0.139–7.852)	0.966	0.998	1.679 (0.395–7.145)	0.483	0.805
TR	0.658 (0.259–1.671)	0.378	0.748	0.596 (0.265–1.337)	0.209	0.445
MR	3.132 (1.185–8.282)	0.021^*^	0.147	2.619 (1.089–6.299)	0.032^*^	0.181
AR	4.351 (0.997–18.980)	0.051	0.298	3.240 (0.760–13.820)	0.112	0.302
PR	5.927 (1.364–25.760)	0.018^*^	0.147	4.272 (1.004–18.170)	0.049^*^	0.181
PVS	0.529 (0.191–1.471)	0.222	0.598	0.689 (0.297–1.596)	0.384	0.672
BPV	2.545 (0.338–19.130)	0.364	0.748	4.083 (0.959–17.380)	0.057	0.181
PS	0.776 (0.104–5.817)	0.805	0.939	0.561 (0.076–4.148)	0.571	0.869
LSVC	1.239 (0.449–3.418)	0.679	0.849	1.053 (0.385–2.877)	0.921	0.998
AVC	/	0.998	0.998	/	0.998	0.998
RAA	2.361 (0.311–17.930)	0.406	0.748	1.870 (0.250–13.990)	0.542	0.862
ALVOT	/	0.997	0.998	/	0.997	0.998
ARV	/	0.998	0.998	0.782 (0.087–7.047)	0.826	0.997
Cardiomegaly	2.437 (0.952–6.239)	0.063	0.315	2.533 (1.135–5.652)	0.023^*^	0.161
Abnormal location of heart	1.284 (0.462–3.568)	0.632	0.849	1.114 (0.444–2.791)	0.819	0.997
Dextrocardia	0.717 (1.166-3.105)	0.657	0.849	0.522 (0.123-2.213)	0.377	0.672
Mesocardia	1.426 (0.189–10.770)	0.731	0.882	1.014 (0.136–7.534)	0.990	0.998
Levocardia	2.890 (0.665–12.560)	0.157	0.500	3.412 (1.017–11.450)	0.047^*^	0.181
Arrhythmia	1.337 (0.389–4.595)	0.645	0.849	2.822 (1.176–6.772)	0.020^*^	0.161
**Surgical strategy**						
Anatomical repair	1.957 (0.787–4.870)	0.149	0.500	1.511 (0.667–3.422)	0.322	0.626
Physiological repair	0.771 (0.313–1.898)	0.571	0.849	0.871 (0.403–1.881)	0.725	0.976
Fontan	0.262 (0.035–1.964)	0.193	0.563	0.402 (0.095–1.704)	0.216	0.445
Cluster						
Cluster 1	0.578 (0.077–4.334)	0.594	0.849	0.706 (0.159–3.137)	0.647	0.906
Cluster 2	0.577 (0.227–1.468)	0.249	0.623	0.519 (0.231–1.165)	0.112	0.302
Cluster 3	1.980 (0.796–4.925)	0.142	0.500	2.183 (0.980–4.862)	0.056	0.181
**Multivariate**						
SV dysfunction	2.545 (0.479–13.529)	0.273	0.273	1.268 (0.266–6.054)	0.766	0.766
PAH	3.138 (1.013–9.718)	0.047^*^	0.118	3.113 (1.195–8.109)	0.020^*^	0.053
AVSD	36.637 (9.110–147.334)	<0.001^*^	0.005^*^	17.089 (4.024–72.578)	<0.001^*^	0.008^*^
MR	2.222 (0.668–7.391)	0.193	0.241	2.276 (0.792–6.543)	0.127	0.203
PR	3.891 (0.784–19.326)	0.097	0.162	2.504 (0.492–12.757)	0.269	0.307
Cardiomegaly				1.709 (0.681–4.292)	0.254	0.307
Levocardia				4.052 (0.995–16.503)	0.051	0.102
Arrhythmia				3.293 (1.255–8.640)	0.015^*^	0.053

* *Indicated statistical significance*.

*Benjamini-Hochberg method was adopted to p*.

*ALVOT, abnormal left ventricular outflow tract morphology; ARV, abnormal right ventricle morphology; AR, aortic regurgitation; ASD, atrial septal defect; AVC, abnormal vena cava morphology; AVSD, atrioventricular septal defect; BMI, body mass index; BPV, bicuspid pulmonary valve; LSVC, persistent left superior vena cava; MR, mitral regurgitation; PAH, pulmonary arterial hypertension; PDA, patent ductus arteriosus; POF, patent foramen ovale; PR, pulmonary regurgitation; PS, pulmonary artery stenosis; PVS, pulmonary valve stenosis; RAA, right aortic arch; SV, systemic ventricle; SVEDD, systemic ventricular end diastolic diameter; SVEF, systemic ventricular ejection fraction; TR, tricuspid regurgitation; VSD, ventricular septal defect*.

**Figure 3:**
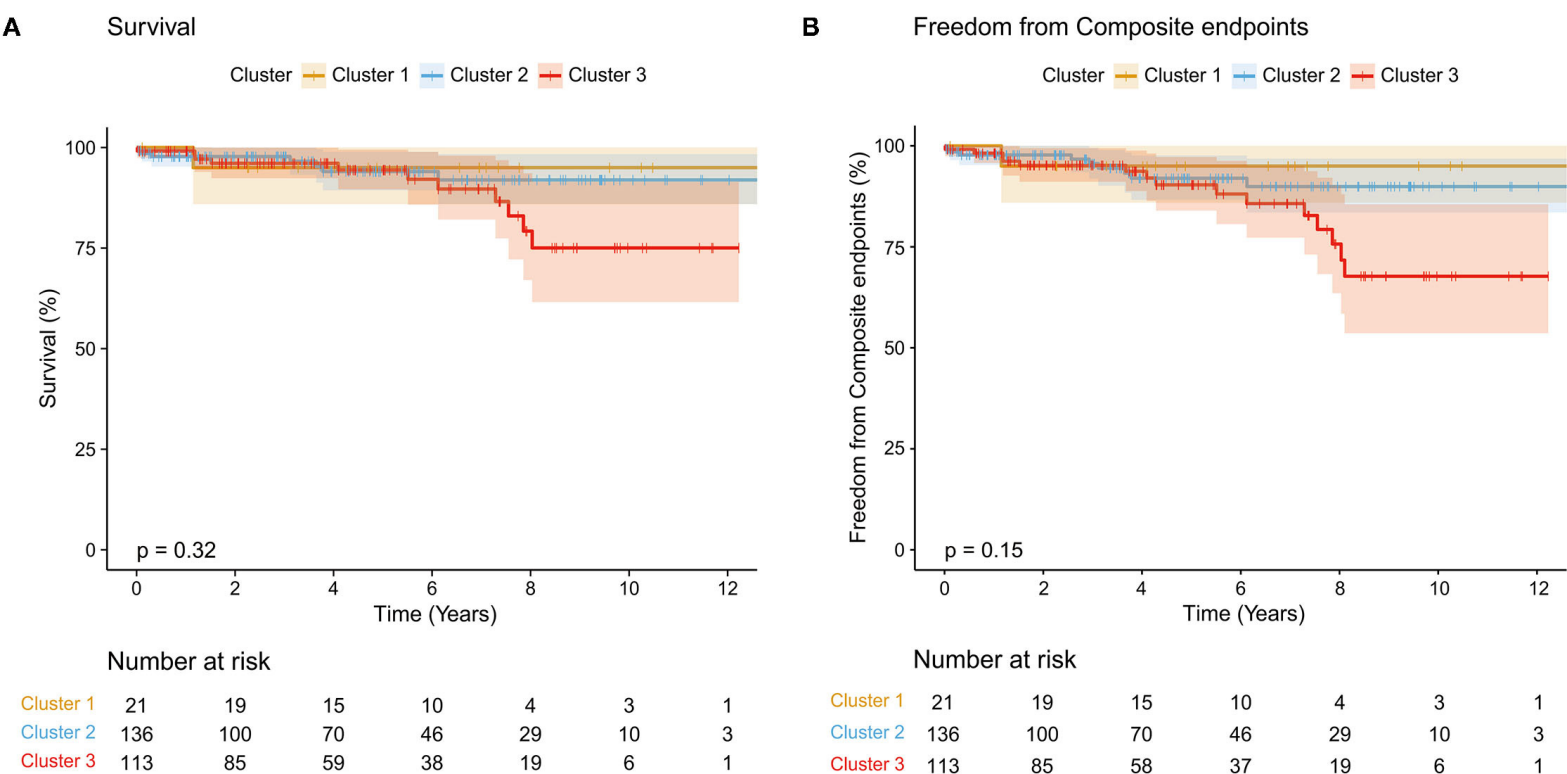
Kaplan-Meier analysis of three clusters. **(A)** Comparison of survival rate among three clusters (*p* = 0.32); **(B)** Freedom from composite endpoints among three clusters (*p* = 0.15). Shading indicates a 95% CI.

**Figure 4:**
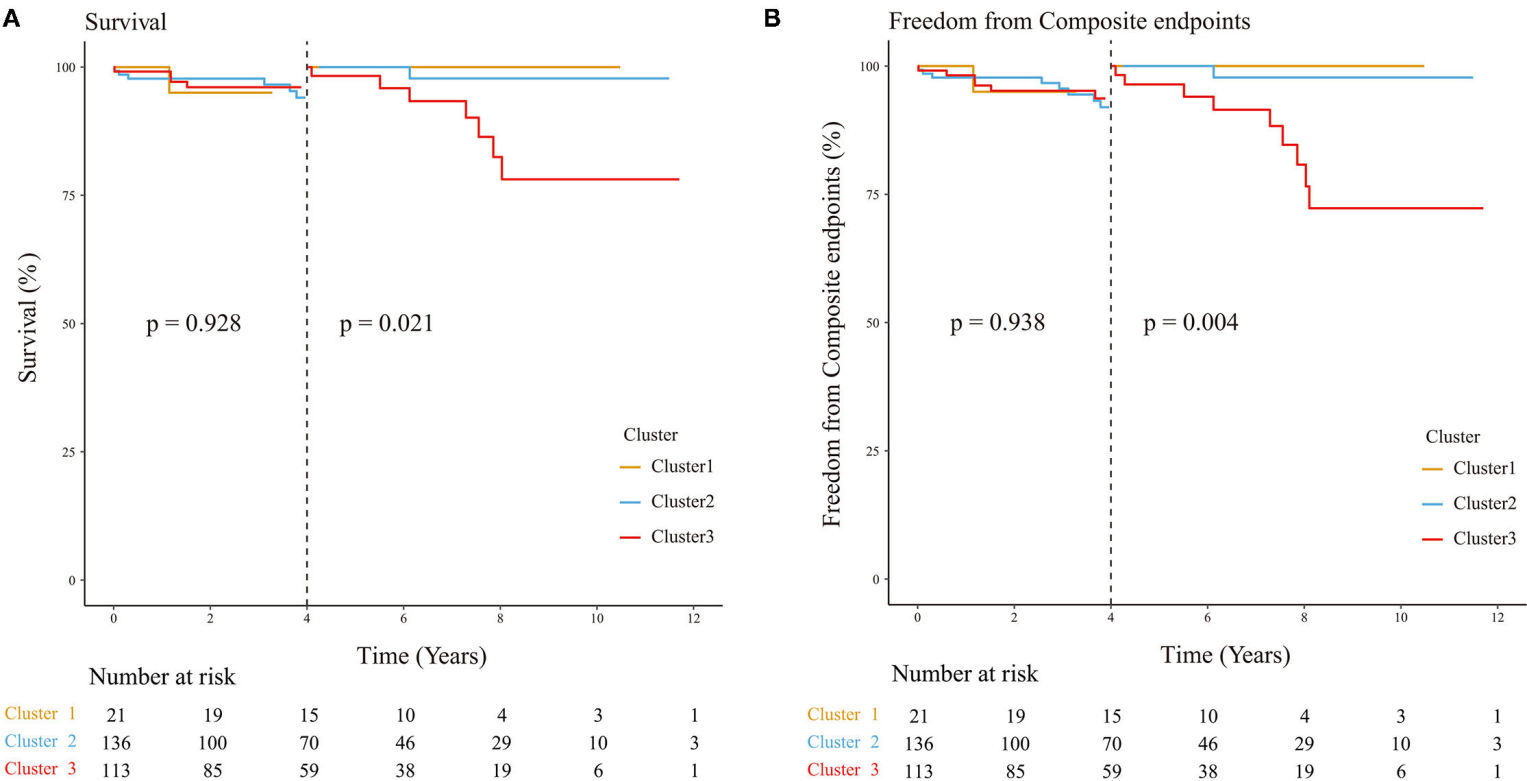
Landmark analysis of three clusters. **(A)** Landmark analysis for survival rate among three clusters; **(B)** Freedom from composite endpoints among three clusters. The landmark analyses reveal statistical significance among three clusters (*p* = 0.021 for overall survival rate, *p* = 0.004 for composite endpoints).

Next, we analyzed the differences in clinical outcomes among the three clusters. As the co-existed phenotypes of patients in three clusters varied, they inevitably led to different physiological conditions that might eventually affect their survival status and increase the risk of suffering from adverse events. We found that there was no significant difference between the three clusters in the overall survival rate (*p* = 0.32, Figure 3A) and composite endpoints (*p* = 0.15, Figure 3B). However, we noticed that different trends began at about 4 years postoperatively for the occurrence of composite endpoint events. Thus, we performed a landmark analysis and found that patients in cluster 3 had significantly worse mid-term outcomes compared with cluster 1 and cluster 2 (*p* = 0.021 for overall survival rate, *p* = 0.004 for compound endpoints; Figures 4A,B).

Finally, we analyzed the impact of surgeries on patients and their interaction with phenotypes. Surgical strategies were significantly varied in the three clusters (*p* < 0.001), which might affect the results. For the whole cohort, surgery did not affect the overall survival rate or composite endpoints (*p* = 0.18 for overall survival rate, *p* = 0.35 for composite endpoints; Supplementary Figures 4A,B) while it dramatically contributed to the risk of reinterventions (*p* = 0.0015) and this mainly occurred in cluster 2 (*p* = 0.039; Supplementary Figures 5A,B, 6A,B). We then compared the outcomes of patients receiving the same surgical strategy in three different phenotypic clusters and found that cluster 3 had a worse mid-term outcome compared with cluster 1 and cluster 2 when surgery was limited to physiologic repair (Supplementary Figures 7A,B, 8A,B, 9A,B), which was almost consistent with the results of comparing outcomes of three phenotypic clusters irrespective of surgery (Figures 3A,B).

## Discussion

Wide-range of co-existed anomalies are still the conundrum in ccTGA population of this era and cause socio-economical burden owing to abroad age divergence of this population (18–20). In this study, for the first time, we comprehensively summarized the cardiovascular phenotypes of patients with ccTGA and analyzed the effects of these combined phenotypes contributing to clinical outcomes integrated with different surgical strategies. We found that patients with more complex phenotypes had significantly worse mid-term prognosis, and surgery also had different effects on the prognosis of patients with different phenotypes.

Being confronted with great heterogeneity in anatomy, hemodynamics, and electrophysiology, the presentation, course, management, and outcomes are not only determined by ccTGA but also other co-existed anomalies (22). Most patients with ccTGA have at least one or more cardiac phenotypes, and these phenotypes significantly affect the natural course of the disease (2). In our cohort, a total of 58 phenotypes were identified, and most patients were presented with three or more different additional phenotypes (Figures 1, 2B). PAH, AVSD, MR, PI, cardiomegaly, and levocardia are risk factors of either death or composite endpoints (Table 2). As a common concern for ccTGA population, TR did not contribute to survival or reintervention, which was consistent with the reports from other centers (23, 24). However, in a previous report from our center, severe TR was associated with the composite endpoints, such as death, heart transplantation, and congestive heart failure (25). The discrepancy may be because we did not limit the degree of TR in this study. In addition to structural abnormalities, the arrhythmic burden of patients with ccTGA, i.e., paroxysmal supraventricular tachycardia, atrial arrhythmia, and complete atrioventricular block, was high and increased with time (26). Even for patients with isolated ccTGA, the occurrence of arrhythmia was reported as one of the important factors affecting the natural history (22). Our study consistently showed that arrhythmias were significantly attributable to patients' outcomes (*p* = 0.015). Collectively, various phenotypes in the ccTGA population are crucial elements in patient prognosis evaluation.

In previous studies, VSD and left ventricular outflow tract obstruction were the major co-anomalies considered for patient grouping and they had not focused on effects of phenotypes that were less influential or clinically considered to be less symptomatic. However, there is no consensus about which phenotypic combinations may have unexpected consequences for patients. As a comprehensive and widely used database of human phenotypes, HPO provides us an inspiring tool for clustering patients with phenotypic heterogeneity, considers comprehensive phenotypes of a patient as far as possible, and presents a relatively complete physiological state of patients' cardiovascular system, which may serve as a tool for phenotype-based risk stratification. Regarding the results of our study, patients of cluster 3 were revealed significantly different mid-term prognosis compared with the other two clusters. In cluster 3, a fair number of patients have unique phenotypic combinations, and several specific phenotypes only presented in one patient, which made a phenotypically extremely heterogeneous group. This group could not be defined by a few specific phenotypes, so they considered a group of patients with a high-risk phenotypic combination. In fact, it does not contradict our goal, which was to identify patients at high risk, rather than focusing on the correlation of a few phenotypes with patient outcomes. Grouping patients by phenotypic similarity through HPO can partially eliminate the influence of phenotypic factors, which may also help us make a phenotypically bias-free cohort for exploring the association between prognosis and other factors, such as surgery.

Due to the divergence and complexity of the anatomical structure of ccTGA, the optimal surgical treatment has not reached a consensus (27). Physiological repair and anatomical repair are two major surgical strategies of ccTGA, while several palliations are prone to acceptable clinical outcomes, such as the Fontan procedure. For maintaining a normal morphologic right ventricular function, the focus of surgical treatment selection was shifted from physiological repair to anatomical repair, but the survival rate after anatomic repair varied among different studies (28). After taking phenotypic factors into account, we found that there was a significant difference in surgical strategy among the three clusters. The selection of treatment was essentially determined by different physiological conditions resulting from different phenotypes of patients, so it was reasonable that operations for different groups of patients varied. However, we found that there was no significant difference in the occurrence of death and heart failure of patients who received different surgical treatments. Consequently, we speculated that the different outcomes of patients in the three phenotypic clusters were caused by phenotypes themselves instead of surgery. For patients with physiological repair, a significant difference in the mid-term prognosis of patients with different phenotypes was observed. We postulated that adopted types of physiological repair operations were determined by the different physiological states of patients, which was also an indirect consequence of the phenotypes. For the patients of cluster 2, different surgical strategies caused a significant difference in prognosis that anatomic repair increased the risk of reintervention in comparison with physiological repair and Fontan palliation (Supplementary Figures 5A,B). VSD and PVS were distinctive characteristics of cluster 2, which was equivalent to the mainstream patient population included in most studies. For patients with both VSD and pulmonary stenosis, Fontan is a feasible option when anatomical risk factors impede biventricular repair, and it has achieved a satisfactory mid-term outcome (29). Anatomical repair of ccTGA sacrificed short-term prognosis but improved long-term prognosis so that it is associated with significant early mortality and morbidity (28, 30). Therefore, for patients in cluster 2, more attention should be paid to choosing the appropriate surgical procedure.

If a large cohort is available, we assume that accurate phenotypic risk stratification can be performed based on the patient's disease profile. Hence software based on such algorithms may be promising for patient risk stratification. According to our experience and data, most of the patients with ccTGA after surgery met satisfactory therapeutic effect, thus some patients did not follow the medical advice for periodic revisits. It may be possible to avoid the occurrence of unexpected adverse events if we can accurately identify patients with high-risk phenotypes and inform them in advance. However, our preliminary exploration of the novel phenotypical risk stratification modality in 270 patients with ccTGA needs to be verified in future studies with different ethnicity and genetic background, and larger cohorts.

Our study had several other limitations: due to the nature of the retrospective single-center study, ethnic genetic background, morbidity, and hospital treatment decisions might be biased. For HPO-based clustering, although all cardiovascular phenotypes had been considered, the severity of each phenotype was neglected, for instance, mild, moderate, and severe TR. For patients with pulmonary atresia, single ventricle, and double outlet right ventricle resulting in complex physiological conditions, ccTGA may not be the main diagnosis at this time and the received treatment might be discrepant, so they were excluded from this study.

## Conclusion

Diversely co-existed anomalies of patients with ccTGA are the major culprit in prognosis evaluation and HPO-instructed clustering delineates a novel phenotypic risk stratification strategy that might beneficially improve prognosis prediction and clinical decision of the ccTGA population.

## Data Availability Statement

The raw data supporting the conclusions of this article will be made available by the authors, without undue reservation.

## Ethics Statement

The studies involving human participants were reviewed and approved by Ethics Committee of Fuwai Hospital. Written informed consent for participation was not provided by the participants' legal guardians/next of kin because: This is the retrospective study.

## Author Contributions

QH and HS contributed to the conceptualization, methodology, EMR reviewing, follow-up, data analysis, and manuscript writing. ZZ contributed to the supervision, conceptualization, professional suggestion, and revision. SL contributed to EMR reviewing, supervision, conceptualization, professional suggestion, and revision. XS contributed to the investigation and follow-up. WC did the investigation. YW worked on graphing optimization. RL provided professional revision suggestions and grant for the study. All authors contributed to the article and approved the submitted version.

## Funding

The study was supported by CAMS Innovation Fund for Medical Sciences (CIFMS, 2020-I2M-C&T-B-061, RL), CAMS Initiative for Innovative Medicine (CAMS-I2M) (no. 2016-I2M-1-016, ZZ), the National Natural Science Foundation of China (no. 82070326, ZZ), and CAMS Innovation Fund for Medical Sciences (CIFMS, 2020-I2M-C&T-A-009, SL).

## Conflict of Interest

The authors declare that the research was conducted in the absence of any commercial or financial relationships that could be construed as a potential conflict of interest.

## Publisher's Note

All claims expressed in this article are solely those of the authors and do not necessarily represent those of their affiliated organizations, or those of the publisher, the editors and the reviewers. Any product that may be evaluated in this article, or claim that may be made by its manufacturer, is not guaranteed or endorsed by the publisher.

## Supplementary Material

Supplementary Figure 1 Flowchart for the whole study. Electronic medical records from 2009 to 2020 were reviewed and 380 patients were obtained initially. After excluding complex phenotypes that might affect the results and patients lost to follow-up, a total of 270 patients were enrolled for downstream analysis eventually. DORV, double outlet right ventricle; HPO, human phenotype ontology; PA, pulmonary atresia; SV, single ventricle.

Supplementary Figure 2 Ontology diagrams of HPO terms for each cluster. **(A)** Cluster 1; **(B)** cluster 2; **(C)** cluster 3. The circles with borders indicate phenotypes presented in each cluster respectively (the phenotypes absent in the HPO database are not shown). The shade of color indicates the frequency of terms. Arrows indicate the relationship between terms and subitems. HPO, the human phenotype ontology.

Supplementary Figure 3 Distribution of phenotype combinations. Phenotypic combinations that occurred in more than two patients are shown (since the population of cluster 1 is small, we demonstrate all the combinations), **(A)** cluster 1, **(B)** cluster 2, and **(C)** cluster 3. AALH, abnormal anatomic location of the heart; ACAM, abnormal coronary artery morphology; AF, atrial fibrillation; AR, aortic regurgitation; ASD, atrial septal defect; AVB, atrioventricular block; CAF, coronary artery fistula; ccTGA, congenitally corrected transposition of the great arteries; CoA, coarctation of aorta; LBBB, left bundle branch block; LSVC, persistent left superior vena cava; MR, mitral regurgitation; PAC, premature atrial contraction; PAT, paroxysmal atrial tachycardia; PAH, pulmonary arterial hypertension; PI, pulmonary insufficiency; POF, patent foramen ovale; PS, pulmonary arterial stenosis; PVS, pulmonary valve stenosis; RVD, right ventricular dilation; TR, tricuspid regurgitation; VSD, ventricular septal defect; VE, ventricular extrasystoles; VT, ventricular tachycardia.

Supplementary Figure 4 Kaplan-Meier analysis of three surgical strategies. **(A)** The comparison of survival rate among three surgical strategies (*p* = 0.18); **(B)** Freedom from composite endpoints among three surgical strategies (*p* = 0.35). Shading indicates a 95% CI.

Supplementary Figure 5 Reintervention rate of three surgical strategies. **(A)** Freedom from reintervention among three surgical strategies (*p* = 0.0015); **(B)** Effects of different surgical strategies on reintervention in patients of cluster 2 (*p* = 0.039). Shading indicates a 95% CI.

Supplementary Figure 6 Kaplan-Meier analysis of different surgical strategies in cluster 1 and cluster 3. **(A)** Freedom from reintervention of different surgical strategies in cluster 1 (*p* = 0.95); **(B)** Freedom from reintervention of different surgical strategies in cluster 3 (*p* = 0.064). Shading indicates a 95% CI.

Supplementary Figure 7 Kaplan-Meier analysis of physiological repair group among three clusters. **(A)** The comparison of survival rate in physiological repair group among three clusters (*p* = 0.84); **(B)** Freedom from composite endpoints in physiological repair among three clusters (*p* = 0.21). Shading indicates a 95% CI.

Supplementary Figure 8 Kaplan-Meier analysis of anatomical repair group among three clusters. **(A)** The comparison of survival rate of anatomical repair among three clusters (*p* = 0.74); **(B)** Freedom from composite endpoints of anatomical repair among three clusters (*p* = 0.85). Shading indicates a 95% CI.

Supplementary Figure 9 Kaplan-Meier analysis of Fontan group between cluster 2 and 3. **(A)** The comparison of survival rate of Fontan procedure between cluster 2 and cluster 3 (*p* = 0.21); **(B)** Freedom from composite endpoints of Fontan procedure between cluster 2 and cluster 3 (*p* = 0.65). No patient received the Fontan procedure in cluster 1. Shading indicates 95% CI.

## Abbreviations

ccTGA: congenitally corrected transposition of the great arteries
HPO: human phenotype ontology
VSD: ventricular septal defect
AVSD: atrioventricular septal defect
PAH: pulmonary arterial hypertension
TR: tricuspid regurgitation
PVS: pulmonary valve stenosis
SVEF: systemic ventricular ejection fraction
SVEDD: systemic ventricular end diastolic diameter
MR: mitral regurgitation
PI: pulmonary insufficiency
EMR: electronic medical records.
