# Supplementary material for: Cardiovascular Phenotypes Profiling for L-Transposition of the Great Arteries and Prognosis Analysis
Source: Front Cardiovasc Med. 2022 Jan 21;8:781041. doi: 10.3389/fcvm.2021.781041 (PMC8814104; doi:10.3389/fcvm.2021.781041)
Supplement: Supplementary file 1 [file Table_1.DOCX]

**Table E1. 58 HPO terms for coding cardiac phenotypes**

| HPO term | HPO name | Abbreviation |
| --- | --- | --- |
| HP:0011540  HP:0001629  HP:0005180  HP:0001642  HP:0001631  HP:0001655  HP:0001640  HP:0001653  HP:0002092  HP:0001651  HP:0001678  HP:0001643  HP:0004415  HP:0005301  HP:0011599  HP:0012020  HP:0005110  HP:0010444  HP:0005182  HP:0001659  HP:0006682  HP:0006695  HP:0010316  HP:0001718  HP:0011670  HP:0010954  HP:0004383  HP:0004756  HP:0011713  HP:0004763  HP:0001704  HP:0004971  HP:0006699  HP:0010446  HP:0011560  HP:0011640  HP:0004927  HP:0012304  HP:0001680  HP:0011669  HP:0005133  HP:0011703  HP:0006671  HP:0004749  HP:0011709  HP:0004307  HP:0011103  HP:0001707  HP:0005345  HP:0001679  HP:0010438  HP:0001633  HP:0001702  HP:0001646  HP:0011641  HP:0006704  HP:0001711  HP:0011675 | Congenitally corrected transposition of the great arteries  Ventricular septal defect  Tricuspid regurgitation  Pulmonic stenosis  Atrial septal defect  Patent foramen ovale  Cardiomegaly  Mitral regurgitation  Pulmonary hypertension  Dextrocardia  Atrioventricular block  Patent ductus arteriosus  Pulmonary artery stenosis  Persistent left superior vena cava  Mesocardia  Right aortic arch  Atrial fibrillation  Pulmonary insufficiency  Bicuspid pulmonary valve  Aortic regurgitation  Ventricular extrasystoles  Atrioventricular canal defect  Ebstein anomaly of the tricuspid valve  Mitral stenosis  Left superior vena cava draining to coronary sinus  Hypoplastic right heart  Hypoplastic left heart  Ventricular tachycardia  Left bundle branch block  Paroxysmal supraventricular tachycardia  Tricuspid valve prolapse  Pulmonary artery hypoplasia  Premature atrial contractions  Tricuspid stenosis^*^  Mitral atresia^*^  Single coronary artery origin^*^  Pulmonary artery dilatation^*^  Hypoplastic aortic arch^*^  Coarctation of aorta^*^  Left superior vena cava draining directly to the left atrium^*^  Right ventricular dilatation^*^  Sinus tachycardia^*^  Paroxysmal atrial tachycardia^*^  Atrial flutter^*^  Atrioventricular dissociation^*^  Abnormal anatomic location of the heart  Abnormal left ventricular outflow tract morphology  Abnormal right ventricle morphology  Abnormal vena cava morphology  Abnormal aortic morphology  Abnormal ventricular septum morphology  Abnormal mitral valve morphology  Abnormal tricuspid valve morphology^*^  Abnormal aortic valve morphology^*^  Coronary artery fistula^*^  Abnormal coronary artery morphology^*^  Abnormal left ventricle morphology^*^  Arrhythmia | ccTGA  VSD  TR  PVS  ASD  POF  Cardiomegaly  MR  PAH  Dextrocardia  AVB  PDA  PS  LSVC  Mesocardia  RAA  AF  PI  BPV  AR  VE  AVSD  EA  MS  LSVCCS  HRHS  HLH  VT  LBBB  PST  TVP  PAHP  PAC  TS  MA  SCAO  PAD  HAA  COA  LSVCLA  RVD  ST  PAT  AFR  AD  AALH  ALVOTM  ARVM  ASVCM  MAPCAs  MVSA  AMVM  ATVM  AAVM  CAF  ACAM  ALVM  Arrhythmia |

*Appeared in only one patient

Abbreviations: HPO, human phenotype ontology.

**Table E2. The phenotypes that differed among the three groups**

| Phenotypes | All (n=270) | Cluster1 (n=21) | Cluster2 (n=136) | | Cluster3 (n=113) | p-value |
| --- | --- | --- | --- | --- | --- | --- |
| ASD  VSD  POF  TR  AR  PVS  PI  PAH  PS  Cardiomegaly  VT  LBBB  AF  VE  PAC | 82(0.3)  194(0.72)  48(0.18)  128(0.47)  5(0.02)  115(0.43)  6(0.02)  40(0.15)  17(0.06)  41(0.15)  3(0.01)  3(0.01)  8(0.03)  5(0.02)  2(0.01) | 1(0.05)  0(0)  0(0)  16(0.76)  1(0.05)  1(0.05)  2(0.1)  8(0.38)  1(0.05)  10(0.48)  2(0.1)  2(0.1)  5(0.24)  3(0.14)  2(0.1) | 54(0.4)  127(0.93)  31(0.23)  45(0.33)  0(0)  92(0.68)  0(0)  0(0)  1(0.01)  9(0.07)  0(0)  1(0.01)  1(0.01)  0(0)  0(0) | 27(0.24)  67(0.59)  17(0.15)  67(0.59)  4(0.04)  22(0.19)  4(0.04)  32(0.28)  15(0.13)  22(0.19)  1(0.01)  0(0)  2(0.02)  2(0.02)  0(0) | | 4.69E-04  1.97E-22  1.56E-02  4.06E-06  2.72E-02  1.18E-17  6.96E-03  3.84E-14  1.12E-04  5.79E-06  7.73E-03  1.65E-02  5.12E-05  8.62E-04  5.78E-03 |

Abbreviations: ASD, atrial septal defect; VSD, ventricular septal defect; POF, patent foramen ovale; TR, tricuspid regurgitation; AR, aortic regurgitation; PVS, pulmonic stenosis; PI, pulmonary insufficiency; PAH, pulmonary arterial hypertension; PS, pulmonary artery stenosis; VT, ventricular tachycardia; LBBB, left bundle branch block; AF, atrial fibrillation; VE, ventricular extrasystoles; PAC, premature atrial contractions.

**Table E3. Survival rate**

|  | 30 days | 1 years | 5 years | 10 years |
| --- | --- | --- | --- | --- |
| Overall | 99.26% (98.24%-100%) | 98.5% (97.05%-99.97%) | 94.29% (91.11%-97.57%) | 85.4% (78.59%-92.79%) |
| Cluster 1 | 100% (100%-100%) | 100% (100%-100%) | 95% (85.91%-100%) | 95% (85.91%-100%) |
| Cluster 2 | 99.26% (97.84%-100%) | 97.75% (95.26%-100%) | 94.03% (89.36%-98.94%) | 91.94% (85.95%-98.34%) |
| Cluster 3 | 99.12% (97.4%-100%) | 99.12% (97.4%-100%) | 94.41% (89.62%-99.46%) | 75.04% (61.59%-91.43%) |
| Anatomic | 97.06% (93.12%-100%) | 95.57% (90.78%-100%) | 89.48% (81.67%-98.03%) | 81.63% (69.68%-95.62%) |
| Physiologic | 100% (100%-100%) | 99.33% (98.04%-100%) | 94.72% (90.58%-99.06%) | 86.74% (77.54%-97.05%) |
| Fontan | 100% (100%-100%) | 100% (100%-100%) | 100% (100%-100%) | 94.44% (84.43%-100%) |
