# Supplementary figures and images for: Cardiovascular Phenotypes Profiling for L-Transposition of the Great Arteries and Prognosis Analysis
Source: Front Cardiovasc Med. 2022 Jan 21;8:781041. doi: 10.3389/fcvm.2021.781041 (PMC8814104; doi:10.3389/fcvm.2021.781041)

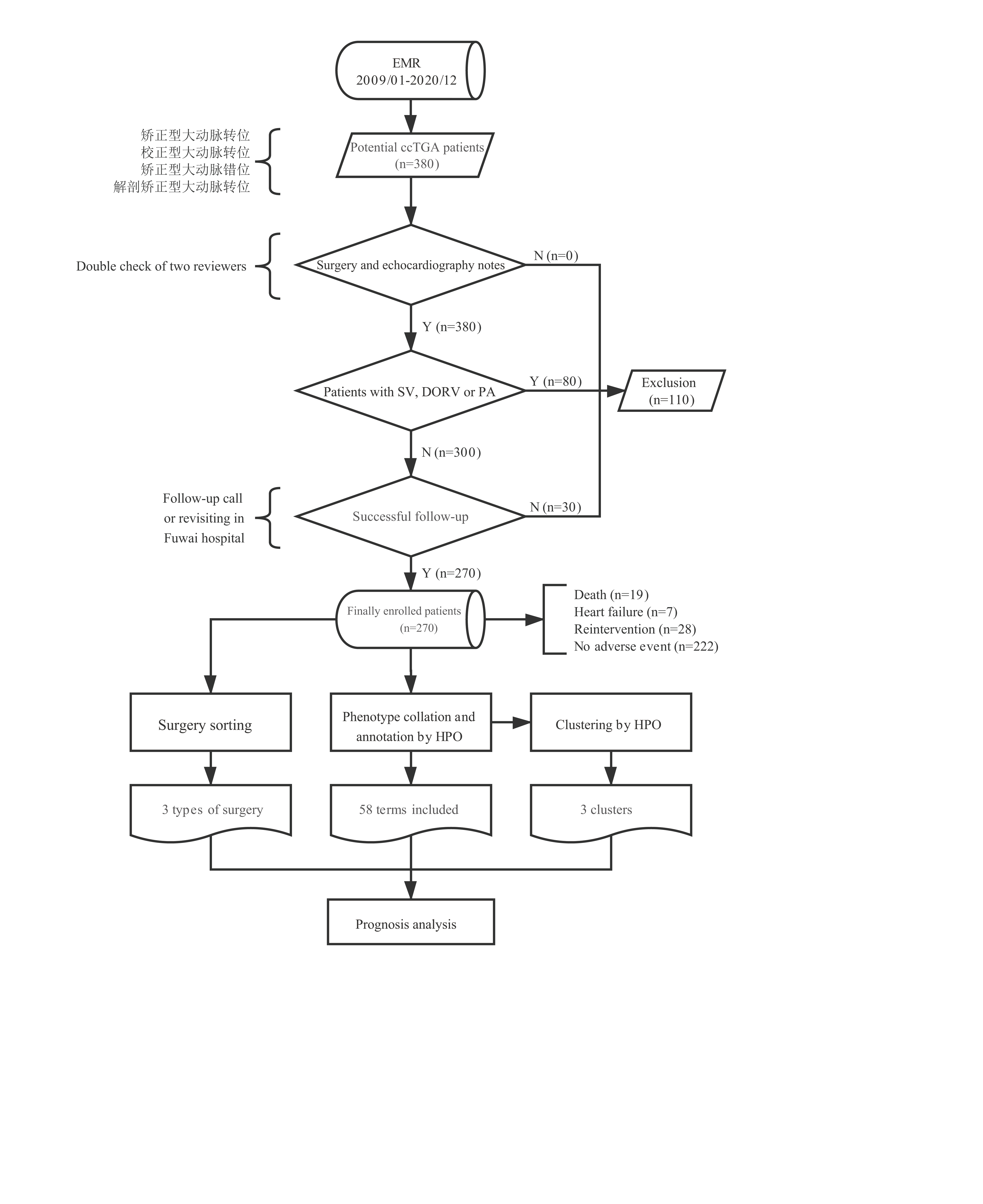

Supplement: Supplementary Figure 1 — Flowchart for the whole study. Electronic medical records from 2009 to 2020 were reviewed and 380 patients were obtained initially. After excluding complex phenotypes that might affect the results and patients lost to follow-up, a total of 270 patients were enrolled for downstream analysis eventually. DORV, double outlet right ventricle; HPO, human phenotype ontology; PA, pulmonary atresia; SV, single ventricle. [file Image_1.JPEG]

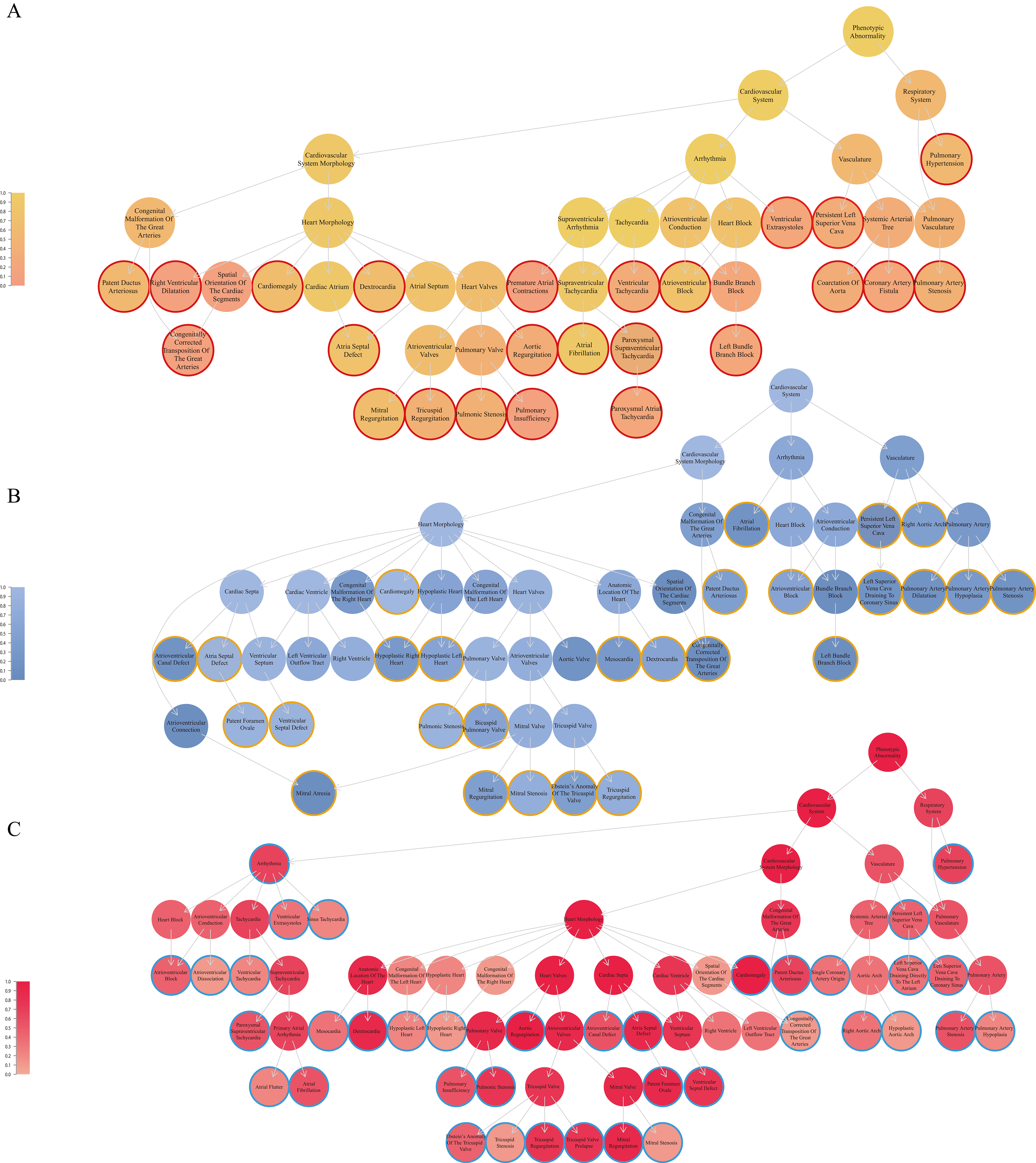

Supplement: Supplementary Figure 2 — Ontology diagrams of HPO terms for each cluster. (A) Cluster 1; (B) cluster 2; (C) cluster 3. The circles with borders indicate phenotypes presented in each cluster respectively (the phenotypes absent in the HPO database are not shown). The shade of color indicates the frequency of terms. Arrows indicate the relationship between terms and subitems. HPO, the human phenotype ontology. [file Image_2.JPEG]

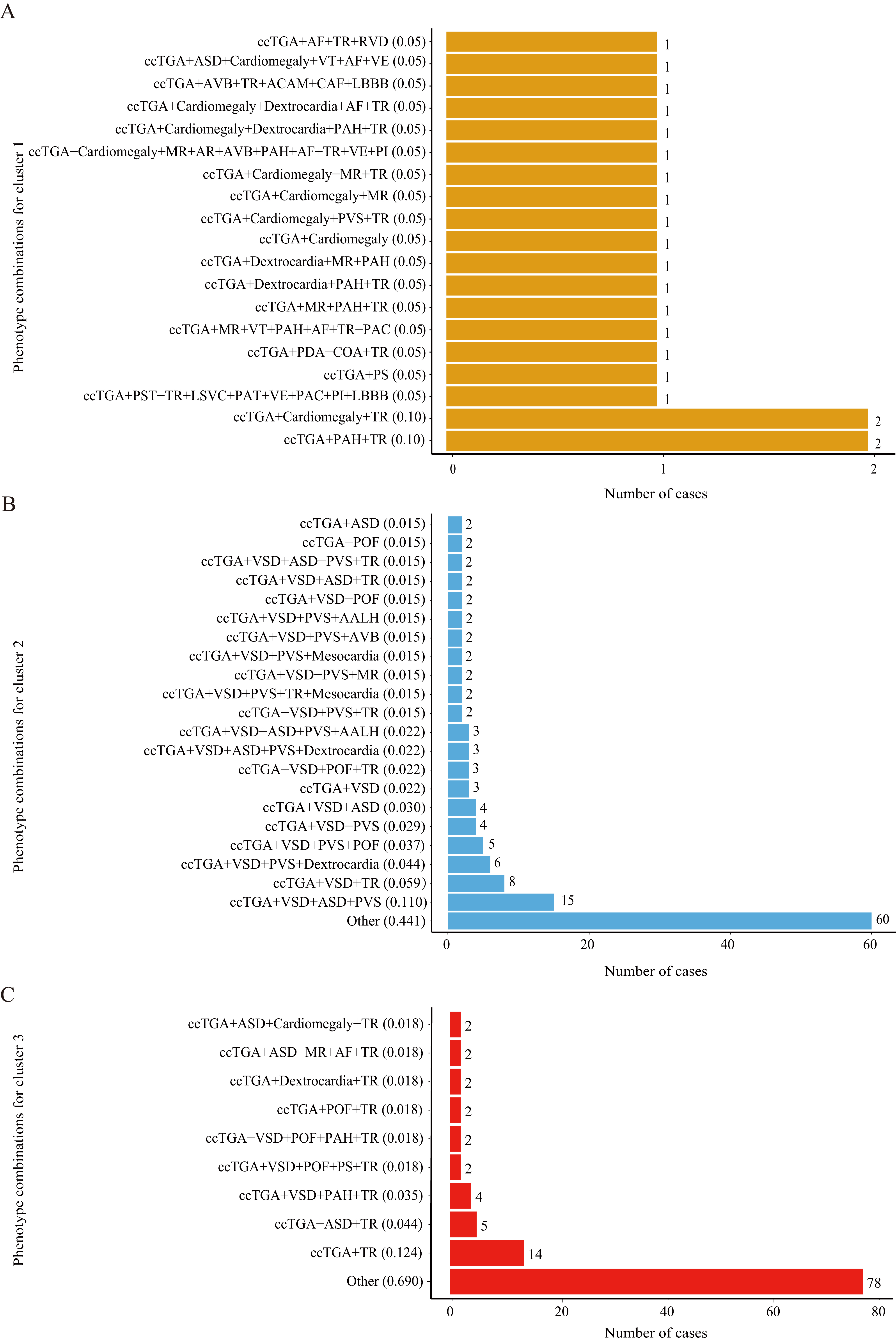

Supplement: Supplementary Figure 3 — Distribution of phenotype combinations. Phenotypic combinations that occurred in more than two patients are shown (since the population of cluster 1 is small, we demonstrate all the combinations), (A) cluster 1, (B) cluster 2, and (C) cluster 3. AALH, abnormal anatomic location of the heart; ACAM, abnormal coronary artery morphology; AF, atrial fibrillation; AR, aortic regurgitation; ASD, atrial septal defect; AVB, atrioventricular block; CAF, coronary artery fistula; ccTGA, congenitally corrected transposition of the great arteries; CoA, coarctation of aorta; LBBB, left bundle branch block; LSVC, persistent left superior vena cava; MR, mitral regurgitation; PAC, premature atrial contraction; PAT, paroxysmal atrial tachycardia; PAH, pulmonary arterial hypertension; PI, pulmonary insufficiency; POF, patent foramen ovale; PS, pulmonary arterial stenosis; PVS, pulmonary valve stenosis; RVD, right ventricular dilation; TR, tricuspid regurgitation; VSD, ventricular septal defect; VE, ventricular extrasystoles; VT, ventricular tachycardia. [file Image_3.JPEG]

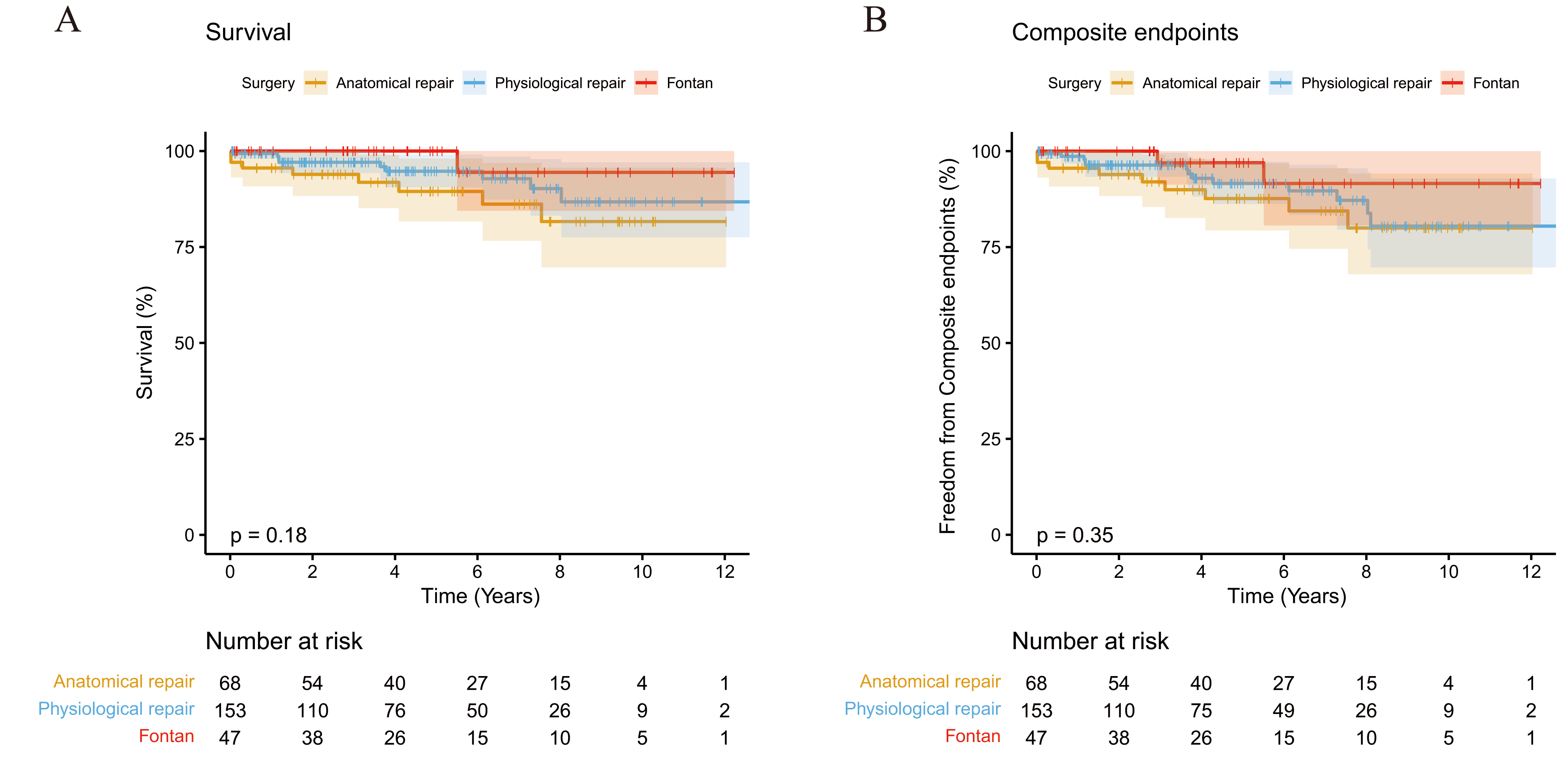

Supplement: Supplementary Figure 4 — Kaplan-Meier analysis of three surgical strategies. (A) The comparison of survival rate among three surgical strategies (p = 0.18); (B) Freedom from composite endpoints among three surgical strategies (p = 0.35). Shading indicates a 95% CI. [file Image_4.JPEG]

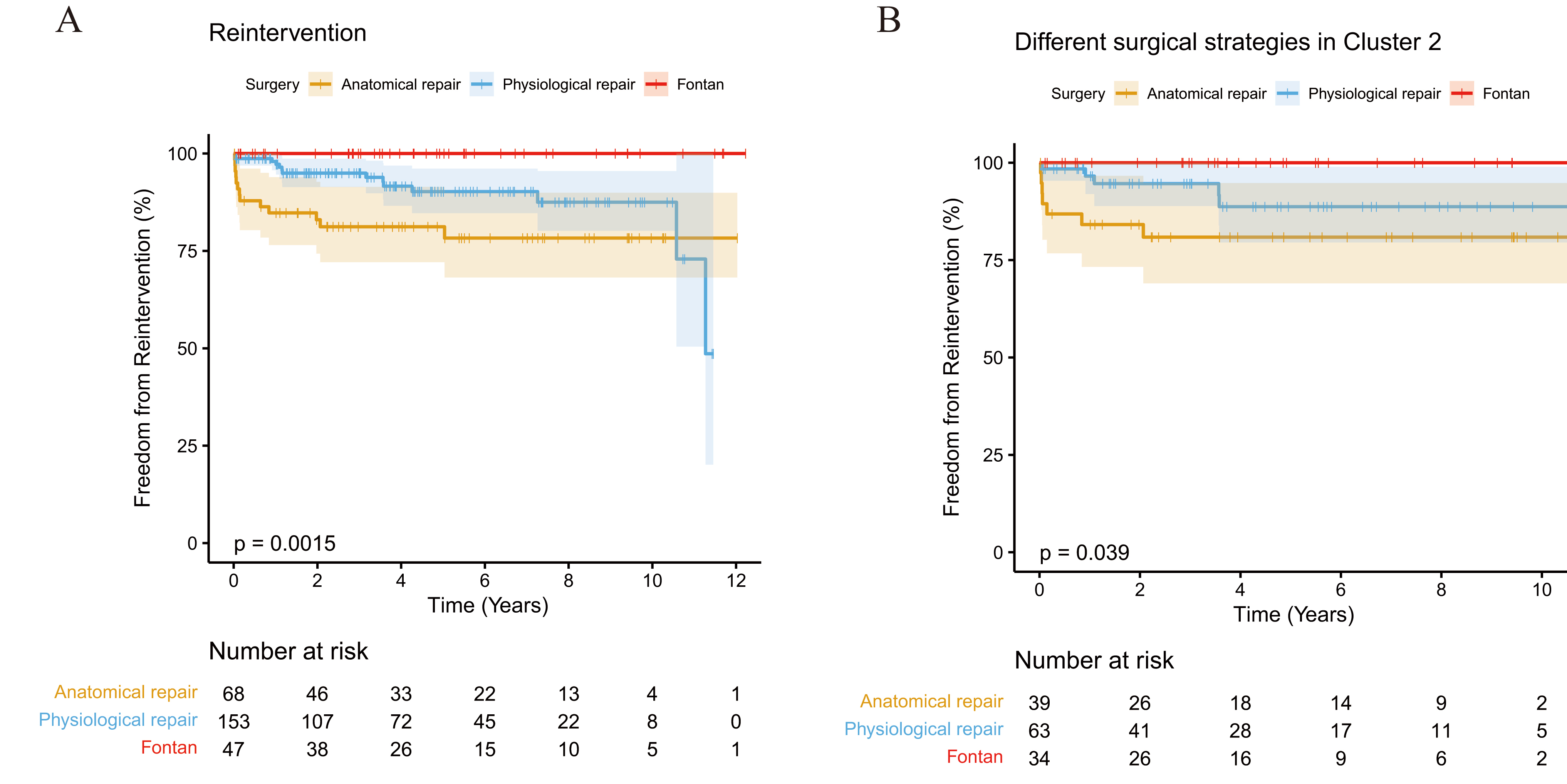

Supplement: Supplementary Figure 5 — Reintervention rate of three surgical strategies. (A) Freedom from reintervention among three surgical strategies (p = 0.0015); (B) Effects of different surgical strategies on reintervention in patients of cluster 2 (p = 0.039). Shading indicates a 95% CI. [file Image_5.JPEG]

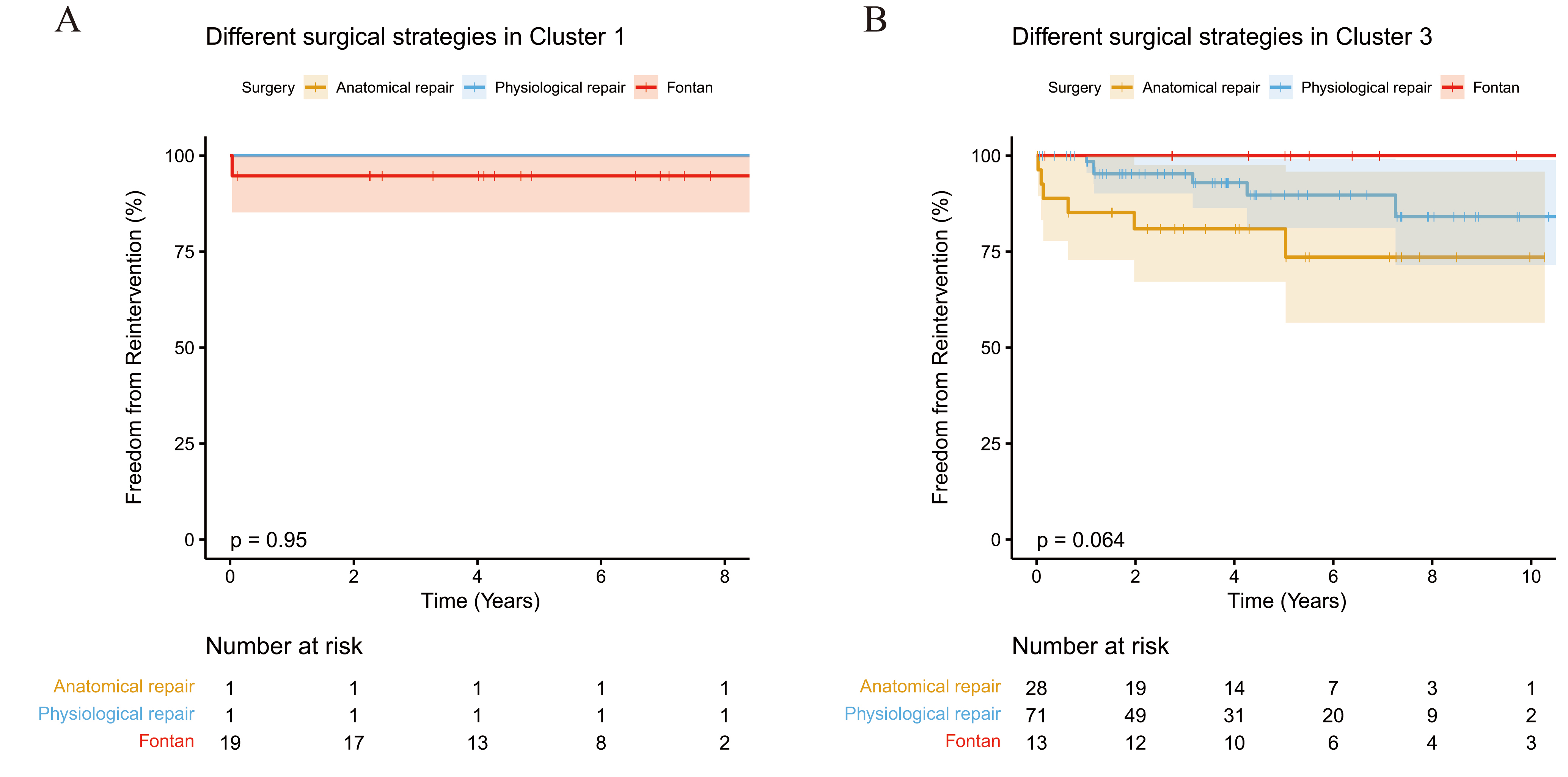

Supplement: Supplementary Figure 6 — Kaplan-Meier analysis of different surgical strategies in cluster 1 and cluster 3. (A) Freedom from reintervention of different surgical strategies in cluster 1 (p = 0.95); (B) Freedom from reintervention of different surgical strategies in cluster 3 (p = 0.064). Shading indicates a 95% CI. [file Image_6.JPEG]

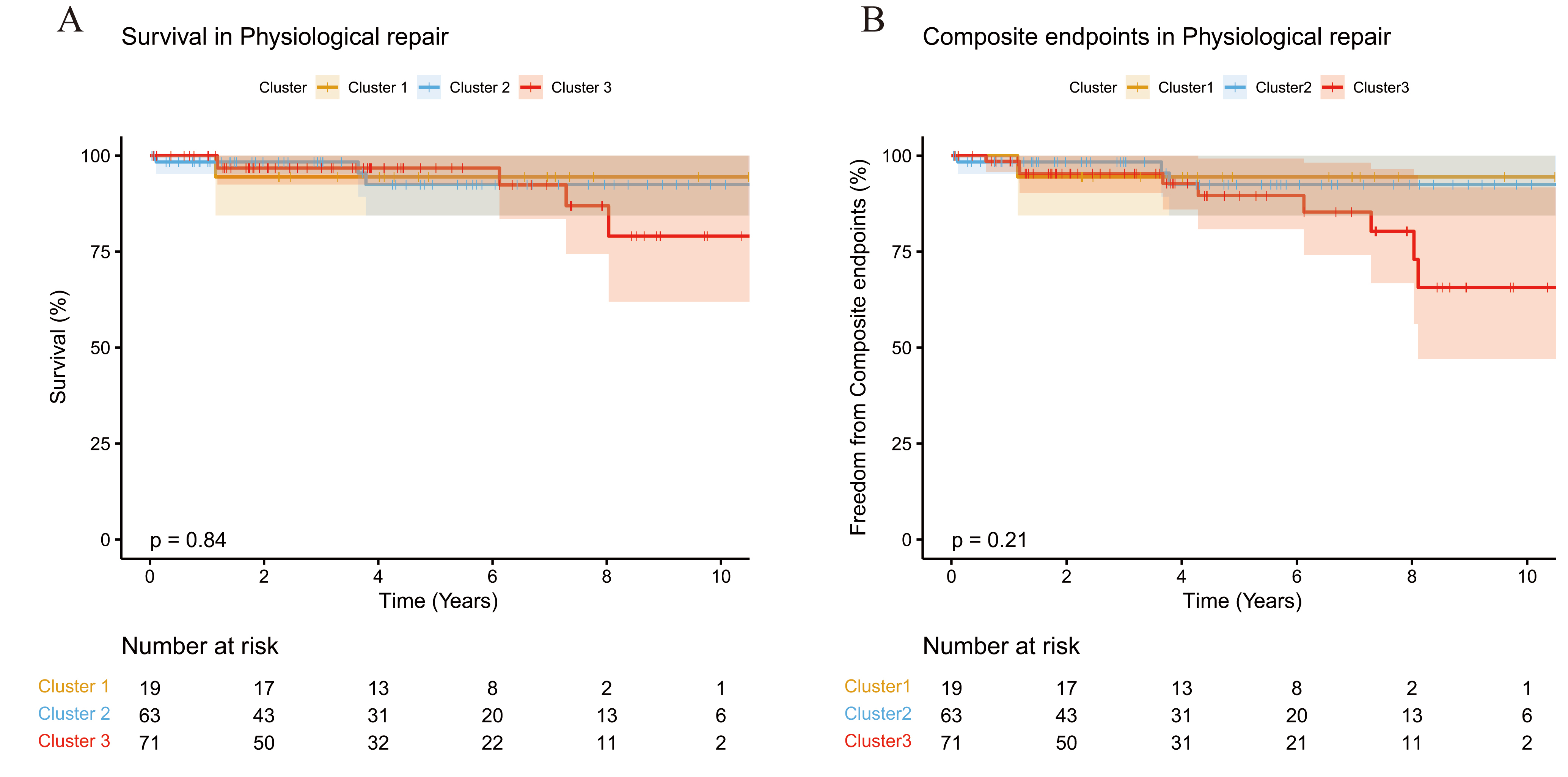

Supplement: Supplementary Figure 7 — Kaplan-Meier analysis of physiological repair group among three clusters. (A) The comparison of survival rate in physiological repair group among three clusters (p = 0.84); (B) Freedom from composite endpoints in physiological repair among three clusters (p = 0.21). Shading indicates a 95% CI. [file Image_7.JPEG]

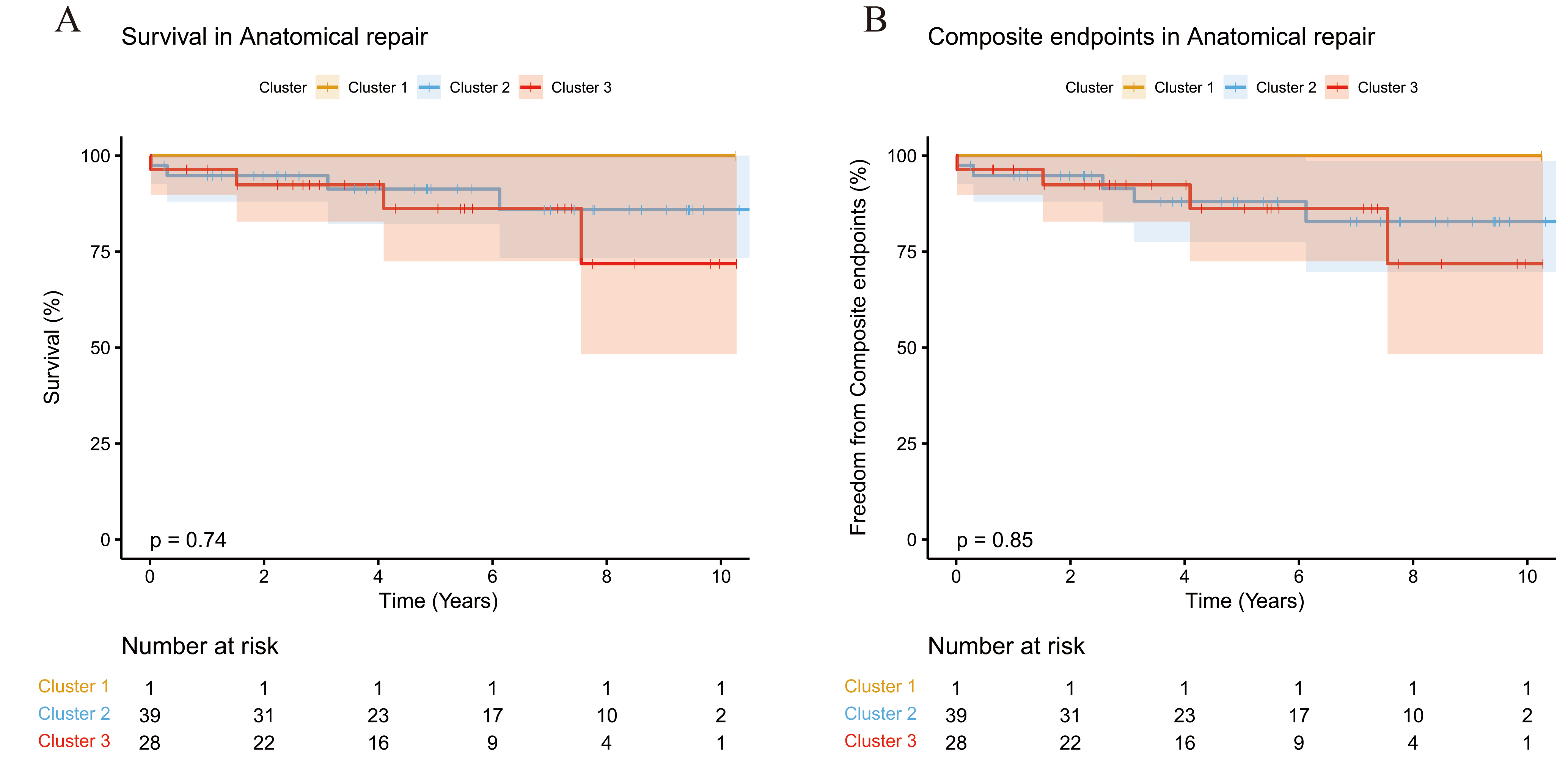

Supplement: Supplementary Figure 8 — Kaplan-Meier analysis of anatomical repair group among three clusters. (A) The comparison of survival rate of anatomical repair among three clusters (p = 0.74); (B) Freedom from composite endpoints of anatomical repair among three clusters (p = 0.85). Shading indicates a 95% CI. [file Image_8.JPEG]

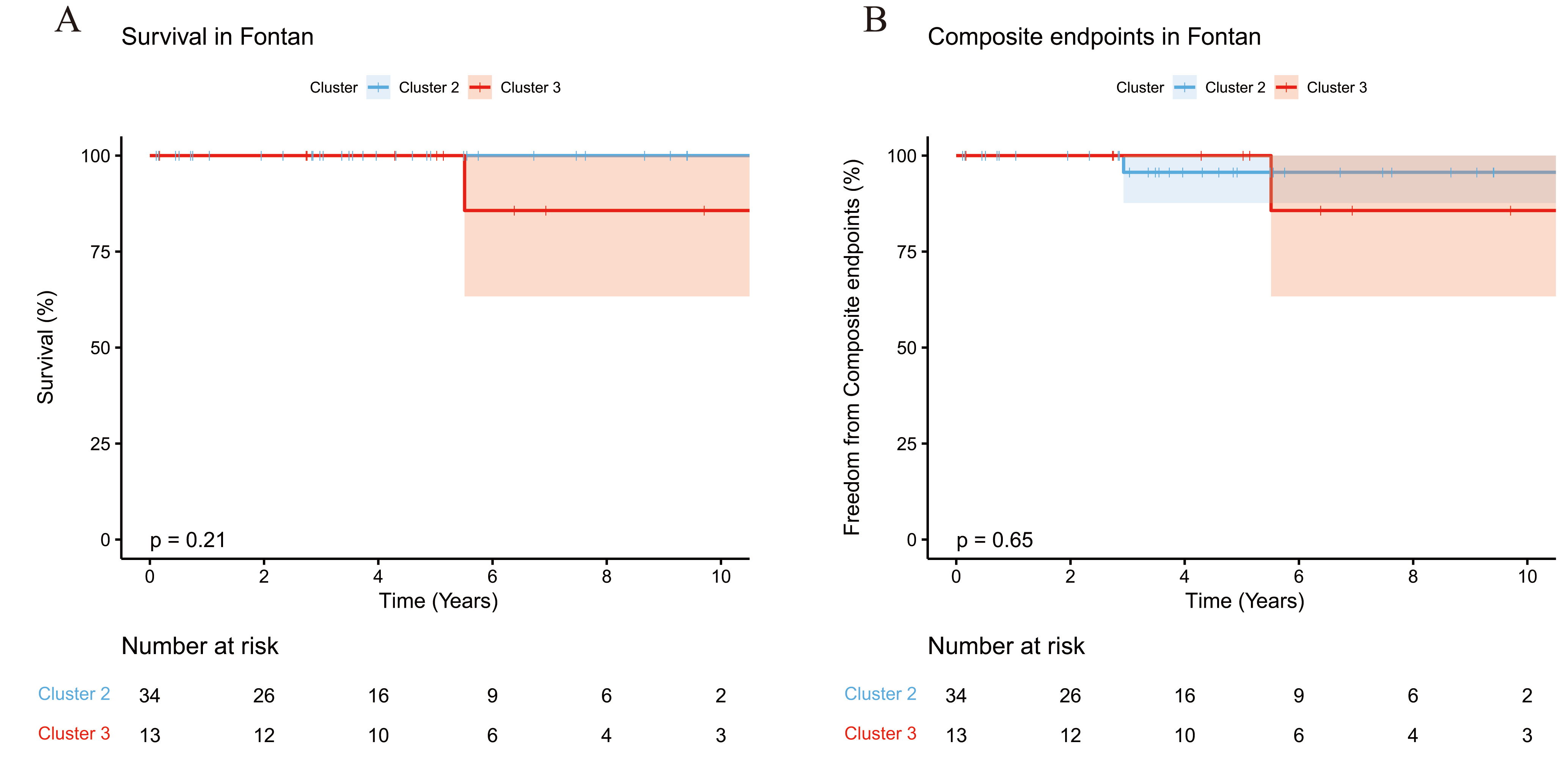

Supplement: Supplementary Figure 9 — Kaplan-Meier analysis of Fontan group between cluster 2 and 3. (A) The comparison of survival rate of Fontan procedure between cluster 2 and cluster 3 (p = 0.21); (B) Freedom from composite endpoints of Fontan procedure between cluster 2 and cluster 3 (p = 0.65). No patient received the Fontan procedure in cluster 1. Shading indicates 95% CI. [file Image_9.JPEG]
